# A novel teaching model using a massive online open course for soft skills development in the veterinary medicine curriculum

**DOI:** 10.3389/fvets.2025.1558473

**Published:** 2025-04-10

**Authors:** Nélida Fernández Pato, María Auxiliadora Ruíz-Rosillo, Lydia Calleja Bueno, Isabel Rodríguez Hurtado, María Dolores Vivas Urias

**Affiliations:** ^1^Facultad de Veterinaria, Universidad Alfonso X el Sabio, Madrid, Spain; ^2^Facultad de Ciencias de la Educación, Universidad Alfonso X el Sabio, Madrid, Spain

**Keywords:** innovation, veterinary, MOOC, soft skills, teaching, learning, Coursera, parasitic diseases

## Abstract

**Introduction:**

Teaching and learning methods in Higher Education are constantly adapting looking for a better achievement of one day competencies in each subject along the Veterinary Medicine Degree. Alfonso X el Sabio University is developing its innovative and pedagogical model, entirely centered on the students and based on the development of the most demanded soft skills by employers named “UAX Skill School.” This model has been created by using Coursera platform and selecting online massive open courses (MOOCS) for being adapted to different subjects to ensure the students develop the most useful soft skills. This research summarizes the process and results of the adaptation and implementation of the MOOC “Learning How to Learn” from the Coursera platform in Parasitic Diseases subject which complements and enhances the learning process of Veterinary students.

**Methods:**

During 2022–2023, 2023–2024, and 2024–2025 academic years, the MOOC “Learning How to Learn” has been integrated and adapted in the subject of Parasitic Diseases in 7 practical sessions before students performed the MOOC and achieved certification. Medallia survey data has been conducted to review student insights.

**Results:**

Student participation has been over 90%, the dropout rate is less than 2.8% and the performance and learning experience per academic year have reached. Likewise, the invested time (6.41, 5.09, and 4.19 h respectively) and the global participation 94.16%, as well as the conformity of the students with respect to the learning process with grades from 7.85 before the introduction of the pedagogical model to 8.59 after it.

**Discussion:**

Innovative methodologies centered on technology are valuable for developing soft skills as evidenced by the implementation of the MOOC “Learning How to Learn” within the context of Parasitic Diseases. Key success factors include the adaptation and integration of these resources into the curriculum. Further research is required to attain a comprehensive understanding of the potential impact of this MOOC on the learning outcomes and soft skills development of Veterinary students.

## Introduction

In recent decades, Higher Education has been undergoing efforts to innovate, in other words, to create new approaches to perceive and tackle issues by adapting to current times and the global changes that are taking place (1) are increasingly trying to innovate and change their entrenched pedagogical strategies (2).

However, this pedagogical innovation is much more than employing digital tools or new technologies. Innovation implies adapting or integrating digital tools and methods to address real issues or challenges in a meaningful and context-sensitive way as a product of problem-solving skills, critical thinking and creativity (3).

One of the key points of pedagogical innovation undoubtedly is its ability to change and adaptability. Having adapted to the change of knowledge transfer toward educational systems based on the development of competencies, not only of knowledge, but also of values and behaviors to fulfill the requirements of society (4). In the last 20 years, transversal competencies such as teamwork, leadership, creativity or problem-solving have become very valuable. They have been integrated into the curriculum of the Veterinary Medicine Degree as part of the day one competences that every veterinarian should develop and acquire throughout their university education (5). The development of these competencies is grounded in the application of active methodologies, which place students at the center of the learning process. This shift moves from a teacher-centered model to a learner-centered model. While the teacher remains responsible for imparting knowledge to achieve the competencies described, there is a collaborative relationship between the teacher and the students. Students are no longer passive listeners; they are active learners, allowing them to contribute to the development of learning methodologies and evaluation methods (6). This approach also provides greater flexibility in acquiring competencies, facilitates the long-term consolidation and application of knowledge, and fosters the development of skills such as constructive communication, effective dialog, and critical thinking, thereby promoting more autonomous and lifelong learning (7).

Moreover, the use of active methodologies is closely associated with experiential learning, also referred to as “learning by doing,” a concept popularized by the American philosopher John Dewey. Dewey advocated enhanced learning through active participation and founded an experimental school at the University of Chicago in 1896.

The pedagogical innovation promoted in Higher Education in recent years could be defined as a combination of the search for professionals with updated theoretical knowledge and adapted to a globalized world and capable of performing their profession in multidisciplinary work environments where they can display all the skills acquired or enhanced throughout their university education (8).

Furthermore, there is an increasing academic training among the population. Therefore, unlike what could be assumed in previous times, having a university degree is no longer enough nor does it guarantee a future employment position. For these reasons, greater employability is increasingly sought by training professionals with skills that allow them to lead an interdisciplinary and globalized world, trying to define the skills that every student of the 21st century should have, considering their way of thinking, working, work tools and their adaptability to live and be part of a globalized world (9). In this sense, the forecast on the evolution of the importance of skills development in the next 5 years by the World Economic Forum (2023) places creative thinking in first place, ahead of analytical thinking and technological literacy. Among the 10 most demanded core skills by employers are those related to self-efficacy, such as curiosity and lifelong learning; resilience, flexibility and agility; and motivation and self-awareness, highlighting the importance of people adopting a culture of lifelong learning.

Therefore, in parallel to the disciplinary competencies that involve, the knowledge and understanding of theoretical knowledge in any university degree, students will acquire professional competencies, also known as soft skills.

In Veterinary Medicine Degree programs, the use of active methodologies has significantly enhanced the skills of the students, motivation, and subsequent integration into the job market. Universities and curricula have traditionally focused on developing hard skills or cognitive knowledge, but there is now a growing emphasis on soft skills that are essential for students’ professional careers, regardless of their specific branch of Veterinary.

Notable methodologies include problem-based learning (PBL) and case-based learning (CBL), which are particularly effective in the clinical setting. These approaches help students develop and acquire theoretical and practical competencies such as analysis, evaluation, interpretation, cognitive awareness, and communication skills (10). Additionally, work-based learning provides students with greater opportunities for observation and autonomy, adhering to the principles of self-directed learning (11).

Nevertheless, the implementation of these methodologies should consider modifications to previously applied models to enhance student learning and academic performance (12). In this context, the introduction of flipped learning methodology facilitates the engagement of the students in classroom or clinical activities in a more autonomous manner, under the supervision of the responsible teacher or teachers. This methodology also contributes to the enhancement of critical thinking, personalized learning, in-depth understanding of the subject matter, and the development of collaborative skills, while promoting student agency in their own learning process (13).

The introduction of hybrid models, promoted among other causes by the Covid-19 pandemic, has been facilitating students to combine face-to-face activities with sessions or activities that can be carried out online, promoting student autonomy and the development of competencies necessary in their future professional activity (11). These hybrid models, as well as the pedagogical innovation of recent years, aim to include, in addition to theoretical knowledge, other competencies in students known as soft skills, since students trained in teaching environments that use active methodologies show greater analytical and teamwork skills (14).

For these reasons, a model that lists and defines the competencies veterinarians should have is currently being developed, dividing them into three sections: communicative, business and digital competencies. This incorporates both the Day One Competencies proposed by the European Association of Establishments for Veterinary Education (EAEVE) and those proposed by the European System for the Evaluation of Veterinary Education (ESECT) (15).

In recent years, the advent of various information and communication technologies (ICT) has catalyzed the evolution and transformation of educational models. A notable example is the adoption of Massive Open Online Courses (MOOCs), which facilitate asynchronous student learning at significantly reduced economic cost (16). This learning modality offers numerous advantages, including increased student motivation and the promotion of self-regulation skills (17). However, it is crucial to assess the academic level of students beforehand to mitigate the risk of MOOC dropout, where self-regulation again plays a key role (18).

These MOOCs have been adopted by high school students, undergraduate students, as well as pos graduate and doctoral students (19). In Higher Education, MOOCs have been employed not only for training students but also for training teachers in digital competencies using nano-MOOCs (20). They have also demonstrated potential for utilization within the Veterinary profession (21).

Among the virtual education platforms created to offer various MOOCs, one of the best known is Coursera, founded by Andrew Ng and Daphne Koller, professors of computer science at Stanford University in 2011. This platform currently offers more than 12,000 courses, practical projects, and certificate programs translated into English, Spanish, French, Italian, Chinese, and other languages. These Massive Open Online Courses are considered a supplement to the traditional learning process, aiding not only in the acquisition of competencies but also in self-development, the development of soft skills, and the promotion of self-regulation in the learning process (22).

Educational innovation implies a genuine change in educational practice so that students develop, during their studies, not only essential cognitive skills for their future profession, but also a series of essential competencies that enable them to function in a changing and multidisciplinary work environment in which the use of technology is always required.

For these reasons, Alfonso X el Sabio University (UAX) has developed an educational model that provides a UAX-maker training experience. This model ensures that, during their academic studies in any degree, the students of the Veterinary Medicine Degree develop and certify the five soft skills most in demand by employers (23).

Storytelling: defined as the ability to transmit and modulate a message through active listening to connect with the audience, using the necessary resources to adapt to their characteristics and context. It includes empathy and active listening.

Agile methodologies and teamwork meant the ability to interact, listen with empathy and offer solutions integrating different points of view to collaborate effectively with people from different fields and disciplines in the achievement of common goals. It includes self-efficacy skills: resilience, flexibility and agility.

Analytical thinking: ability to analyze and evaluate information, decompose problems or complex situations and detect patterns to propose solutions and make decisions. It involves the use of computer tools and, therefore, contributes to technological literacy.

Disruptive thinking: ability to generate innovative ideas, products and solutions to complex problems or situations in collaborative and co-creative environments.

Leadership and ethics: capacity to motivate, influence and lead others, fostering their development to collaborate effectively and achieve common goals within a framework of values.

In 2021, UAX launched the UAX Skill School to help UAX students develop the five core skills that define UAX graduates. To do so, UAX chose Coursera as a strategic partner UAX Skill School encourages continuous learning and lifelong learning for graduates.

UAX Skill School integrates Coursera courses throughout its degree programs, providing students with the opportunity to obtain certificates that enhance their employability. For each skill, resources are selected based on the maturity and level of the student, their field of knowledge and the different levels of competency development. The contents and activities are integrated in the planning and evaluation of each subject. Learning can be autonomous or guided, depending on the maturity of the student and the methodology used by the instructor (23).

In the Veterinary Medicine Degree program, a specific course has been selected for each academic year to develop a particular skill. In each course, the professor is responsible for integrating the skill into the curriculum to facilitate its development among students. In this case, the subject chosen in the third year of the Veterinary Medicine Degree is Parasitic Diseases, which integrates the MOOC “Learning How to Learn: Powerful mental tools to help you master tough subjects” to develop the soft skill of disruptive thinking.

The main goal of the MOOC “Learning How to Learn” is to help students develop effective learning strategies, providing them with tools to manage their own learning process more efficiently (metacognition), and to face new challenges and achieve their goals with self-efficacy, thereby promoting a culture of lifelong learning.

The methodological approach adopted by the instructor in the subject to successfully integrate the MOOC and develop the skill is Flipped Learning (24). During the first semester, students engaged in a combination of face-to-face and online learning activities. These activities were related to both the understanding of the MOOC content and its practical application in acquiring the knowledge and developing the competencies of Parasitic Diseases subject. The practical face to face activities were intentionally designed by the instructor to interrelate the MOOC content with the course learning outcomes, complementing the course learning activities with the application of the knowledge and techniques learned in the MOOC. Subsequently, students were encouraged to complete the MOOC online from home to obtain their certificate.

This paper presents how the MOOC “Learning How to Learn” has been integrated into the Parasitic Diseases course, the benefits observed, as well as the level of satisfaction and engagement of the students enrolled from the academic year 2021–22 to 2024–25.

## Materials and methods

This paper includes the data of students enrolled in the annual subject Parasitic Diseases of the third year of the Veterinary Medicine Degree of the Alfonso X el Sabio University in the academic years 21–22, 22–23, 23–24, and 24–25, attached in Table 1.

**Table 1:** Number of students matriculated in Parasitic Diseases by academic year.

Academic year	Number of students
21–22	325
22–23	301
23–24	218
24–25	205

During the academic year 21–22 the new academic model UAX Skill School had not been implemented, so it can be considered as a control group.

### Description of the Coursera course: “Learning How to Learn”

The MOOC “Learning How to Learn: Powerful mental tools with which you can master difficult topics (Learning How to Learn),” is made up for 5 modules dealing with what is learning, fragmentation, procrastination and memory, learning renaissance and how to release your potential and a last module with more resources. The course instructors are Dr. Barbara Oakley and Dr. Terrence Sejnowski. The course has a total duration of 16 h, although it is considered that about 19 h may be required to be completed and consists of videos, optional readings, assignments to be completed, peer review activities, and quizzes to assess the knowledge achieved (25). Since its launch in 2014, it has been one of the most popular MOOCs with nearly 4 million people enrolled worldwide.

To incorporate the MOOC “Learning How to Learn” and successfully develop the skill in the Parasitic Diseases subject, the head professor conducted an in-depth review of the MOOC content, resources and activities prior to its implementation. This review, first, enabled the teacher to identify the MOOC skills and techniques which could be practically applied and, secondly, to choose the most suitable ones according to the specific content and learning outcomes of the Parasitic Diseases subject.

Once this alignment between the MOOC contents and the subject learning outcomes was accomplished, the professor intentionally designed a series of face-to-face activities to interlink them, so that the practical application of the knowledge and techniques learned in the MOOC would complement the subject learning activities. This connection has two proposals: on the one hand, to facilitate the understanding and integration of the various theoretical and practical competencies that the students should acquire and, on the other hand, to help the students understand the importance of completing the MOOC to develop the soft skill.

Finally, the professor reviewed the subject, planning to include the new activities that would integrate the MOOC with the Parasitic Diseases subject. To this end, seven 1-h practical sessions were designed and conducted during the first semester. These practical sessions were carried out before the students had been given access to the MOOC.

To familiarize themselves with the content and deepen their understanding of the concepts and topics discussed, as described below.

In the first session, questions were prompted about learning, study methodologies, and some of the recurrent difficulties they encountered. These questions captured the attention of the students and revealed that not all had a variety of study strategies, as some were based merely on memorization.

The activities included the identification of parasitic agents and their identification according to morphology, life cycle and diagnostic methods. These activities were framed around simple clinical cases aimed at diagnosing which parasite might be represented in an initial image, which could be macroscopic or microscopic.

The second activity involved providing clues to solve clinical cases using a microscopic image that could be examined microscopically.

Another activity required students to determine whether short sentences were correct or incorrect and explain their reasoning.

In addition, activities were carried out to relate theoretical concepts about the general aspects of Parasitic Diseases.

As a final part of each of the seven sessions, a concluding question was asked about which activity they found most useful. This was intended to highlight that everyone has their own learning method, and not all activities are equally engaging or easy for all students in the classroom.

In the second session, the professor started by asking the students if they knew the meaning of procrastination and gave them an example. Next, images and names of parasitic agents were projected, and the students were asked to match them correctly. After 1 min, the images were removed and the students were asked how many pairs they had made, how they could have improved their results, and what they needed to know. After helping them with the answers, the activity was repeated. This exercise was used to explain the Pomodoro technique. Using the movie Inside Out, the concepts of short-term and long-term memory, the importance of spaced repetition in studying, and the importance of sleep were explained. In addition, the connection between long-term memory and abstract thinking as a basis for scientific thinking and creativity was highlighted.

Clinical cases were included to help students relate microscopic parasitic forms to epidemiological and diagnostic aspects. True or false questions were formulated. Color associations were made with specific parasitic agents to highlight their biological cycles or important biotic or abiotic factors. At the end of the activity, students were asked which one of the developed practical activities they considered most useful.

In the third session, a brief five-minute test was conducted in the virtual classroom. After the test, concepts such as illusions of competence, the Einstellung effect, the memory palace, and overlearning were explained. Clinical cases were proposed, and short questions were posed with video projections to answer. To explain the memory palace, four images were projected to illustrate the epidemiological and clinical aspects of various parasites affecting equines that had been studied theoretically: *Eimeria leuckarti*, Anoplocephalids, strongyles, and *Parascaris*.

Macroscopic and microscopic images were projected to reinforce concepts about sizes, diagnostic techniques, and/or the presence of macroscopic parasitic forms in feces. Sentences were distributed to identify errors, which were then explained. The session concluded with a discussion on the most useful aspects of the activities.

In session 4, an experimental activity was conducted with *Gasterophilus* eggs, to which boiling water was added while they were observed and recorded under a microscope. This was accompanied by a series of questions regarding these parasites. Following this activity, discussions on impostor syndrome and the Dunning-Kruger effect were held.

Students were presented with images and tasked with establishing relationships between them, determining the accuracy of sentences, analyzing clinical cases, identifying errors in sentences, and answering multiple-choice questions. At the conclusion of the session, students were asked to identify the most positively valued section.

Session 5 involved inquiries related to concentration, positive and negative preoccupation, and a review of impostor syndrome. Activities were designed to address uncertainties or concepts that students had struggled with in a knowledge evaluation test conducted earlier that week. During the session, key concepts were summarized, clinical cases were proposed, images were correlated, texts with and without errors were analyzed, and evaluative questions were posed.

In session 6, metaphors and analogies were introduced. A new section titled “Popular Phrases” was proposed, where groups were formed to answer common questions posed by pet owners, requiring the deepest possible parasitological knowledge. New clinical cases, diagnostic techniques, a comprehensive list of questions and true and false questions were presented.

In session 7, the memory palace was used again, mixing it with analogies to remember epidemiological aspects of the ascarids that affect domestic carnivores. Clinical cases were analyzed, images were proposed in which they had to choose a specific parasite and give the corresponding explanation why they had made a choice, famous phrases, clinical cases in groups with clues that they had to look for in trays located on the tables, a list of images and true and false questions were made. As in every work, they ended with the activity that had been most useful to them and with images of themselves working at the laboratory.

After session 7, the students were encouraged to complete the MOOC from home (fully online) to get their certification.

## Results

### Results of participation and experience of the student in the MOOC

To evaluate student participation and experience in the MOOC, the data provided by the Coursera platform were taken into consideration in relation to the data of the students enrolled in the Parasitic Diseases subject in the different academic years analyzed.

### Participation by academic year on the Coursera platform

In Table 2 is detailed by academic year the number of students enrolled in the subject Parasitic Diseases, the number of students enrolled in the MOOC on the Coursera platform and the number of students who completed the course. In academic year 22–23 the number of students enrolled was 301, being lower in academic year 23–24 with 218 and lower in academic year 24–25 with 205 enrollments. In total, in three academic years, out of a total of 724 students were enrolled in the subject, 684 students have signed up for the MOOC and 644 have completed it.

**Table 2:** Number of students matriculated in Parasitic Diseases by academic year, number enrolled in Coursera and number completing the course.

Academic year	Number of enrolled students subject	Number of enrolled students Coursera	Number of students with accomplished Coursera MOOC
22–23	301	278	258
23–24	218	207	192
24–25	205	199	194

### Attrition rate and learning performance

Considering the percentage of enrolled students by academic year (Table 3), it can be observed that in all academic years the percentage has exceeded 90%, being course 24–25, the one in which the highest percentage of enrolled students has been detected, with 97.07% of the enrolled students.

**Table 3:** Percentage of students enrolled and with completed course by academic year, average grade, maximum and minimum values and standard deviation.

Academic year	Enrolled percentage	Completed course percentage	Average score	Max-Min	Standard deviation
22–23	92.36	92.81	8.7	10–7.8	0.05
23–24	94.95	92.75	8.7	10–7.8	0.05
24–25	97.07	97.49	8.8	10–7.9	0.05

The average grade obtained is very similar in the three courses, being the same in the first two academic years, 8.7, and 8.8 in the 24–25 course. As can be seen in Table 3.

During each academic year, the marks achieved by the students were classified into those who scored a 10, a mark between 9 and less than 10, a mark between less than 9 and 8, and a mark of less than 8. It can be noticed that in the first two academic years more than 70% of the students 72.87 and 72.92%, respectively, scored a note lower than 9 and up to 8, while in the last year, the highest percentage of students 64.95% obtained a note between 9 and lower than 10 and the percentage of students who obtained the maximum grade increased from 0.39 and 0.52% of previous years to 2.58%. As shown in Table 4.

**Table 4:** Percentage of students with scores of 10, <10–9, <9–8, and <9–8.

Academic year	Score 10	Score < 10–9	Score < 9–8	Score < 8
22–23	0.39	22.48	72.87	4.26
23–24	0.52	23.44	72.92	3.13
24–25	2.58	64.95	26.80	5.67

### Average time to complete the MOOC per academic year

The actual average time that students required to complete the Coursera course has been decreasing over the academic years, starting with an average of 6.41 h, followed by 5.09 h, and finally 4.19 h. Similarly, the maximum hours needed to complete the course have also decreased, with 15.63 h, 14.04 h, and 13.09 h, as shown in Table 5.

**Table 5:** Average hours used to complete the course, maximum, minimum values, and standard deviation per academic year.

Academic year	Average time	Max-Min	Standard deviation
22–23	6.41	15.63–0	2.91
23–24	5.09	14.04–0	2.88
24–25	4.19	13.09–0.02	2.22

### Participation global results reviewed

This section presents the overall data obtained from the integration of the Coursera course in Parasitic Diseases subject. Over the three academic years during which the UAX Skill School in Parasitic Diseases has been implemented, a total of 724 students enrolled in the subject. Of these, 684 students enrolled in the MOOC, and 644 completed it. The MOOC enrollment rate was 94.16%, and the completion rate was 92.87%. It is important to note that these results differ from those shown in previous tables because repeating students (those who did not pass the course) who completed the MOOC are enrolled and counted again in the following academic year. This is because repeating students do not need to retake the MOOC, although they do participate in the activities proposed in the course that are related to the MOOC.

### Veterinary student satisfaction in the subject of parasitic diseases

To determine student satisfaction in the subject, the institutional professor satisfaction survey was implemented before the end of the course using Medallia’s SaaS tool, Medallia Experience Cloud.

The UAX professor satisfaction survey included 10 items, from which 5 were selected: overall satisfaction with the professor, assessment of learning acquired with the professor, connection with professional life, assessment of participatory and collaborative activities, and information provided on learning.

The results are presented as values from 0 to 10, indicating the average of the students’ responses and the percentage of students who responded. These results reflect both the performance of the teacher implementing the UAX Skill School pedagogical model and the overall response rate for the course, which is taught by 5 teachers.

The results obtained for the five selected items were all above 7 points. As shown in Table 6, the evaluation of all items has improved since the 2022–2023 academic year, when the MOOC was integrated into the subject, compared to the 2021–2022 academic year, when no intervention was implemented. The values for the last two academic years are broadly similar, with an improvement of more than half a point when comparing the results of the last two academic years to those of the initial academic year 2021–2022.

**Table 6:** Average rating of students who responded to the satisfaction survey questions by academic year, with a possible rating from 0 to 10.

Questions	21–22	22–23	23–24	24–25
Connection with professional life	8.2	8.72	8.8	9.27
Conducting participatory and collaborative activities	7.93	8.45	8.56	8.56
Information provided about learning	7.7	8.4	8.63	8.64
Acquired learning with the professor	7.88	8.36	8.67	8.59
Global satisfaction	7.85	8.41	8.58	8.59

The relationship of the subject with professional life has been the most valued data of the survey with a lower grade of 8.2 in the academic year 21–22 and a grade of 9.27 in the academic year 24–25.

The second most valued parameter by the students was information provided on learning, which has increased from a 7.93, to an 8.56 in academic years 22–23 and 23–24.

The third question that has scored the highest was learning acquired with the teacher, which obtains the same grade as the Degree of general satisfaction in 24–25 and has been rising in grade as the academic years have passed, except in academic year 23–24 where the Degree of learning acquired by the teacher was 8.67, while in the following year it is 8.56.

The implementation of participative and collaborative activities has also improved the score achieved, moving from 7.93 to 8.56 in academic years 23–24 and 24–25.

It should be kept in mind that in the first academic year 21–22 when the survey was carried out, the UAX Skill School pedagogical methodology had not been introduced. Therefore, the results presented above are comparable.

Regarding the percentage of student participation, it has been lower than 40% in all the surveys carried out, highlighting that the percentage of responses obtained by the teacher in charge of the UAX Skill School methodology was higher than the overall percentage of responses of the subject in all the years analyzed, as can be seen in Table 7.

**Table 7:** Percentages of answers to the questions of the survey, both professor responsible for UAX Skill School, as well as the global number of professors of the subject, by academic year.

Answers percentage	21–22	22–23	23–24	24–25
Professor in charge of UAX Skill School	36.7	29.7	20.8	32.6
Global subject professors	32.6	27.1	14.1	23.5

It is worth noting that the highest participation values were in the 21–22 course with professor answers values of 36.7% and overall, of 32.6%, while the lowest were in 23–24 with professor values of 20.8% and global values of 14.1%.

## Discussion

### Student-centered active methodologies with the use of technology

Innovation in any field of knowledge, but particularly in education, is a necessity where teachers play a fundamental role in ensuring the success of its implementation, since they serve as the guiding thread (26). The transition from teacher-centered teaching to student-centered education has been a challenging process of transition in teaching methodologies that promote the essential role of the student in his or her own learning process, requiring constant learning, promoting collaborative work and demanding self-regulation of his or her learning (7).

The utilization of information and communication technologies (ICT) has allowed the implementation and application of active methodologies, which are now a reality in the educational field at all academic levels, although some teachers still report a lack of training, large class sizes or even consider it a waste of time (27). However, its implementation faces challenges such as the lack of teacher training, the number of students in the classroom or the time invested in the design and development of classroom activities (28).

In fact, the use of technology within pedagogical innovation not only allows the learning experience to continue beyond the classroom but also enables students to review materials, access a greater number of available resources, and spend the necessary time to adequately consolidate their knowledge.

For these reasons, at UAX, pedagogical innovation and teacher training in active methodologies have been constant in recent years, so that currently, they can apply this knowledge and integrate it appropriately into the learning of their students, as well as explain to students the importance of its introduction and the benefits it brings (7).

Indeed, teacher training is essential, as is the creation of groups or channels for sharing innovative teaching proposals among teachers, promoting attendance at teaching or innovation conferences, and the existence of departments that can support them in improving their teaching innovation skills, as this directly impacts their teaching quality and motivation, as well as their students, by promoting their motivation and accelerating learning (4).

It should not be forgotten that the teacher is key in the learning process of the student and that active methodologies are tools that should be employed to transform, adapt and improve the learning process of the student. However, the introduction of changes or innovations itself does not guarantee their effective integration and usefulness in the pedagogical development, since other elements must be considered to ensure the success of the introduction of active methodologies in the classroom, such as suitable and proper knowledge of the content of the subject by the teacher, pedagogical knowledge of teaching and learning methods and techniques and, of course, technological knowledge to use new technologies in the learning process (27).

### Soft skills and MOOCs in veterinary

Nowadays, these active methodologies are focused on the development of competencies that include knowledge, skills or abilities with which specific tasks can be developed, attitudes such as effective capacity in reference to emotions and feelings and, in addition, aptitudes or talents and learning capabilities of the student.

For these skills and abilities development is required the use of pedagogic methodology targeted to the students, who at present, move in a changing and globalized world in which they must constantly adapt (9).

In Veterinary Medicine Degree studies, both the World Organization for Animal Health (WOAH, founded as OIE) and the World Veterinary Association (WSA) and the European Association of Establishments for Veterinary Education (EAEVE) have defined “one day competencies” that recent graduates have acquired during their training. These include the soft skills of working in diversity, analytical thinking, disruptive thinking, ethical leadership and storytelling, which are, moreover, the five soft skills most in demand by companies and employers (5, 23).

For the introduction of these soft skills, the UAX has developed a new pedagogical model based on the use of MOOCs from the Coursera platform, integrated into various subjects throughout the Veterinary Medicine Degree, as mentioned above.

These MOOCs have demonstrated their effectiveness in enhancing the theoretical knowledge and skills of the students who participate in them, promoting self-regulated learning. In addition, they are significant for their ability to establish interactive communities and to encourage curiosity for learning (17, 19, 29, 30).

Despite having detractors who argue that MOOCs have a limited impact on university education, low course completion rates once students enroll, or that they cannot ensure that the certification was achieved by the person who received it, it is clear that MOOCs have a social impact that should be considered as an opportunity to plan, test and validate new educational models and a disruptive approach to education (31).

### MOOC integration in parasitic diseases subject

MOOCs have been used in the Veterinary field as an approach to expand Veterinary Medicine Degree-related knowledge and competencies of prospective future students (20). They have also been used to evaluate their ability to improve student retention of concepts, established relationships with professors, dropout rates, and their effectiveness in creating sustainable educational content applicable to Veterinary studies (29, 32).

However, the novelty introduced in this research is the integration and adaptation of the MOOC “Learning How to Learn,” one of the most popular MOOCs on the Coursera platform (25), into a subject of the Veterinary Medicine Degree. This not only enables the development of disruptive thinking by allowing students to complete the course and get a certification but also involves the development and application of the innovative UAX Skill School pedagogical model.

In fact, this adaptation of the MOOC for learners taking the course has been previously studied (31). For this reason, before the students took the MOOC “Learning to learn,” the professor provided seven 1-h face to face practical sessions to relate the course to the competencies that students in the Parasitic Diseases subject should achieve throughout the academic year.

This approach tackles one of the most common problems with MOOCs: the role of MOOC instructors and the specific feedback required by each learner. Since MOOCs are available to numerous people from diverse backgrounds, feedback can sometimes be very limited (33).

It should be noted that to adapt the MOOC to the course, the instructor first got the course certification. Subsequently, the professor used the results of self-developed surveys conducted in previous years, inquiring about which teaching materials most helped students in their learning about Parasitic Diseases. This information was used to prepare teaching materials that would facilitate the acquisition of both hard and soft skills.

Among all the methodologies and resources used to adapt the MOOC to the course, case-based learning was one of the most valued methods by the learners. It is also one of the most applied teaching methodologies in the early stages of clinical learning, where the instructor must guide the process (11).

Nevertheless, the aspect that most captured the students’ attention was when they were asked about their study techniques and which activity at the end of each task had helped them learn the most. This is a very important aspect because the more student-centered the learning process is, the better and more meaningful the results will be.

For these reasons, it is crucial to stress the importance of placing the learner at the center of the learning process and valuing his or her opinion when using active methodologies. The perceptions of students and teachers about their use in the classroom may differ in some respects (6).

It should also be noted that this approach was applied over seven sessions. Multiple activities were conducted to achieve the optimal and desired learning outcomes, and they were not conducted in the same order. However, similar activities were repeated throughout the same task, as it has been shown that a student’s attention span varies over the duration of a class or teaching activity. This variation does not follow a linear decreasing pattern but is undulating. Therefore, performing similar activities at different times helps to ensure that all students can achieve the intended learning outcomes of the session (34).

### Assessment of the application of MOOCs in parasitic diseases subject

To objectively evaluate the introduction of any change or innovation in teaching, surveys are undoubtedly a widely used resource (7, 35–37).

Regarding the use of MOOCs for the development of soft skills, one of the identified issues is the dropout rate once students enroll in the courses. This can be considered a parameter to measure student interest in the course (21, 32). Other parameters, such as student participation in the MOOC or feedback between students and instructors, can also be chosen when analyzing the results of their employment (38).

### Attrition and certification rates

During the three academic years analyzed, retention and success rates were measured. These were understood as the percentage of enrolled students compared to the number of students registered on the course, and of those, the number who completed the MOOC and obtained the certificate. The enrollment percentage in any of the three academic years was over 90%: 92.36, 94.95, and 97.07%, respectively. The course completion percentage was similar in the first two academic years, 92.81 and 92.75%, and higher in the last evaluated academic year, 97.49%. All obtained data are higher than in other studies (21, 32). It should be noted that the instructor’s follow-up during the time students had access to the platform, the notifications they received, and personalized help to motivate them to complete the course were considered. It should be highlighted that MOOCs typically have higher follow-up and completion rates when taken by students rather than by individuals interested in a specific topic (39). Additionally, as the level of education increases, so does the follow-up and completion of courses (40).

Indeed, to maintain student motivation and ensure they complete the MOOC, learning activities must follow active methodology, and pedagogical tools and learning strategies must be engaging (38). Therefore, the adaptation of the MOOC to the course is considered a success. Furthermore, student performance improves as they take more courses and enhance their experiences with MOOCs. The students in this study have already completed two of these courses in their first and second years (40).

### Student performance in the MOOC

The average grade obtained by students was 8.7 out of 10 in the first two academic years analyzed, slightly higher in the last academic year with the same maximum and minimum grades. Notably, students improved their grades in the last academic year analyzed, with over 70% of students in previous academic years scoring between 8 and less than 9, and 64.95% scoring between 9 and less than 10, with an increase in the percentage of students scoring a 10, reaching 2.58%.

### Time allocated for MOOC completion and certification

Another parameter analyzed was the number of hours students dedicated to completing the course and obtaining certification. A decrease in the average hours used was detected, from 6.41 h, 5.09 h to 4.19 h in successive academic years, with a maximum number of hours decreasing from 15.63, 14.04 to 13.09 h, although the course indicates approximately 19 h for learning with a flexible schedule. The fact that it is not an excessively long course may favor its completion. However, regarding the course duration and student follow-up and completion, there is little literature, and existing studies have found heterogeneous results (38).

### Student satisfaction UAX skill school pedagogical model

For the evaluation of the student’s perception of their learning in the course, the development of skills and competencies and the active methodologies implemented, the results of five items of the institutional survey of learning with the professor, applied before the end of the course, were analyzed.

The scores resulting from the item “connection of the course with professional life” increased, considering that the lowest score was in the academic year prior to the implementation of the UAX Skill School model, with 8.2. In the 3 years analyzed in which the model had been introduced, the score continued to improve year after year, going from 8.72 in the first year, to 8.8 in the second, and to 9.27 in the last year analyzed.

These results demonstrate that the use of MOOCs and active methodologies is positive. Besides, with experience, the professor responsible for adapting the MOOC to the course progresses and improves his or her teaching methodology, using “tips” from the MOOC to increase his or her teaching, such as the language usage and humor (25).

Moreover, the use of active methodologies has been well received by students, like other studies (8) who perceive differences in teaching between various university courses and express their preferences, unlike what has been observed in another research (7).

Maybe because of the generalized opinion that the subject has a wide connection with professional life and the use of active methodologies, it is evident that the question about the performance of participative and collaborative activities was expected to be well valued by the students, as it did. The lowest grade again was before the change of pedagogical model where the grade was 7.93 and then in the first year of the new model it went to 8.45 and in the following 2 years to 8.65.

This improvement in the grade can be explained by the integration of activities in groups that tried to respond to the students’ demand to learn through applied knowledge and using problem-based learning or the development of mind maps to favor their self-regulated learning but guided by the teacher (41).

It should be considered that not all students answer in the same manner to collaborative activities in the classroom. In some cases, it has been observed that they prefer to work individually, which may need to be studied to improve their evaluation in this aspect. In addition, more activities of this type should be considered throughout the academic year.

The third question of the satisfaction survey referred to the information provided by the professor in relation to learning. In this case, the score increased from 7.77 in the initial year to 8.40, 8.63, and 8.64 in successive years. At this point, the instructor uses active methodologies, short videos to identify errors, exam corrections through practical activities and the preparation of activities with digital resources or the use of gamification to address critical learning points. These methods increase student motivation and can strengthen soft skills, promoting greater integration of knowledge and competencies (42).

In this regard, the professor in charge has perceived that students respond very positively in the classroom when the reasons for employing teaching techniques are explained. Some of these techniques appear in the MOOC used in the course, such as analogies, metaphors or the use of videos to enhance students’ learning (25), as well as other teaching techniques learned during their pedagogical training.

In the fourth question, students replied to their satisfaction with the learning acquired from the professor. Again, the score obtained after the introduction of the UAX Skill School was higher than in the previous course. Specifically, the score was 7.88 in the course without the new model and 8.36, 8.67 and 8.59 in the subsequent courses.

Considering that this question allows students to evaluate various measurable and non-measurable items, it was found that the most important factor for student satisfaction was the subjective perception of well-being or the inclusion of activities that support student autonomy. The explanatory capacity of the professor was highlighted as very relevant, followed by the empathy of the professor, related to his or her concern that all students acquire the requested competencies and skills (43).

The scores obtained suggest that the teaching attitudes of the professor reflect teaching competencies that promote critical thinking, the application of appropriate methodologies to their students, bidirectional communication, collaborative and participatory work, and the implementation of innovative teaching methodologies to improve the quality of teaching (44).

The global satisfaction of students, as in previous answers, has improved with the introduction of the UAX Skill School, going from 7.85 before this model to 8.41, 8.58 and 8.59 in successive academic years. This, although positively valued, implies that there is still time for improvement and to strive for maximum academic and teaching excellence for the students.

### Detected benefits by professor

The benefits observed include an increased connection between students and faculty, as practical activities that adapt the course to theoretical knowledge have facilitated students to find their own learning methods. Students appreciate not only the theoretical or practical knowledge of the subject taught by the professor, but also his or her general and specific pedagogical skills, as well as his or her practical teaching skills (45).

The fact that the professor responsible for introducing the MOOC-related activities was explaining the rationale for doing so, based on scientific articles and the MOOC itself, was very well received by the students.

In fact, adapting the MOOC in each assignment, noting that each activity pursued a specific objective, and ending each assignment by asking which activity had helped them the most, was a very successful approach. Not only did it promote learning by doing, but it placed the learner at the center of their learning process. By understanding what each student needs in each case and how they learn best, teaching can be improved.

Further, it has improved academic results and student satisfaction with the course and activities. It should be kept in mind that the students in the course each year are different, so a longer-term study is needed to observe and measure the results more objectively.

In addition, the MOOC is not only transferable to a course but also allows the development of skills and competencies that can be used in the future, which enables students to learn more easily in the future. It allows faculty to adapt courses to new generations of learners and adjust their teaching materials for better understanding. It also encourages faculty to review the most difficult points or topics in their syllabus and to seek a more practical approach to reduce understanding issues for students.

However, there are some points to note, such as integrating MOOCs as a method for acquiring soft skills that require time outside the classroom and planning the hours students will need outside the classroom to complete the course. Some students find the activities useful but do not understand the importance of soft skills certification, which requires awareness and effort on the part of the faculty members during the process.

Providing a tool where the professor can track the progress of the assigned MOOC facilitates the individual student’s commitment and encourages all students to complete the course and obtain the certification. It should also be kept in mind that the introduction of MOOCs has required attending to the necessities of the students outside the classroom to solve questions and concepts that are not fully understood, which implies the availability of the professor outside of class hours.

## Conclusion

In summary, the adaptation of the MOOC “Learning How to Learn” and its integration into the Parasitic Diseases course in the Veterinary Medicine program has proven to be highly beneficial. This positive impact is reflected not only in the feedback of the students, as evidenced by survey results, but also in the strengthened relationship between professors and students. This innovation fosters a more favorable, open, and multidisciplinary personalized learning environment, which significantly boosts motivation and creativity.

The promising outcomes of this study underscore the transformative potential of integrating MOOCs into traditional curricula. By bridging the gap between conventional teaching methods and modern educational technologies, a dynamic and engaging learning experience is created, preparing students for the complexities of their future careers. Enhanced interaction between students and professors cultivates a collaborative atmosphere, encouraging critical thinking and problem-solving skills essential for Veterinary practice.

Given these encouraging results, it is imperative to extend the analysis with further research. Future studies should aim to deeply evaluate and refine the methodological changes introduced, ensuring the adaptation of the MOOC continues to enhance the acquisition of certified soft skills among university students, particularly future veterinarians. This ongoing evaluation will help solidify the role of the MOOC in advancing Veterinary education and preparing students for their professional careers. By continuously improving and adapting educational approaches, it is possible to ensure that students are not only knowledgeable but also innovative and adaptable professionals ready to meet the challenges of the Veterinary field.

## Data Availability

The original contributions presented in the study are included in the article/supplementary material, further inquiries can be directed to the corresponding author.
